# Computational drug repositioning through heterogeneous network clustering

**DOI:** 10.1186/1752-0509-7-S5-S6

**Published:** 2013-12-09

**Authors:** Chao Wu, Ranga C Gudivada, Bruce J Aronow, Anil G Jegga

**Affiliations:** 1 Division of Biomedical Informatics, Cincinnati Children's Hospital Medical Center, Cincinnati, USA; 2 Department of Computer Science, College of Engineering and Applied Science, University of Cincinnati, Cincinnati, USA; 3 University of Pittsburgh Medical Center, Medical Informatics Technology, Pittsburgh, USA; 4 Department of Pediatrics, College of Medicine, University of Cincinnati, Cincinnati, USA

## Abstract

**Background:**

Given the costly and time consuming process and high attrition rates in drug discovery and development, drug repositioning or drug repurposing is considered as a viable strategy both to replenish the drying out drug pipelines and to surmount the innovation gap. Although there is a growing recognition that mechanistic relationships from molecular to systems level should be integrated into drug discovery paradigms, relatively few studies have integrated information about heterogeneous networks into computational drug-repositioning candidate discovery platforms.

**Results:**

Using known disease-gene and drug-target relationships from the KEGG database, we built a weighted disease and drug heterogeneous network. The nodes represent drugs or diseases while the edges represent shared gene, biological process, pathway, phenotype or a combination of these features. We clustered this weighted network to identify modules and then assembled all possible drug-disease pairs (putative drug repositioning candidates) from these modules. We validated our predictions by testing their robustness and evaluated them by their overlap with drug indications that were either reported in published literature or investigated in clinical trials.

**Conclusions:**

Previous computational approaches for drug repositioning focused either on drug-drug and disease-disease similarity approaches whereas we have taken a more holistic approach by considering drug-disease relationships also. Further, we considered not only gene but also other features to build the disease drug networks. Despite the relative simplicity of our approach, based on the robustness analyses and the overlap of some of our predictions with drug indications that are under investigation, we believe our approach could complement the current computational approaches for drug repositioning candidate discovery.

## Background

Drug development in general is time-consuming, expensive with extremely low success and relatively high attrition rates. To overcome or by-pass this productivity gap and to lower the risks associated with drug development, more and more companies are resorting to approaches, commonly referred to as "*Drug Repositioning*" or "*Drug Repurposing*". Drug repositioning is nothing but identifying and developing new uses for existing or abandoned pharmacotherapies [1]. Since the starting point is usually approved compounds with known bioavailability and safety profiles, proven formulation and manufacturing routes, and well-characterized pharmacology, repositioned drugs can enter clinical phases more rapidly and at a fraction of costs incurred in the discovery and development of completely novel compounds [2]. This new indication discovery has already yielded several successes that include the repositioning of sildenafil from an anti-angina drug to erectile dysfunction treatment and repositioning thalidomide, a withdrawn drug, for leprosy and multiple myeloma. Indeed, it is not surprising that in recent years, repositioned drugs account for ~30% of the new medicines that reach their first markets. Although there are several advantages, rational drug repositioning poses formidable challenges primarily because the molecular basis and the underlying mechanisms of most diseases and drug actions are either elusive or poorly understood, intricate, or are not readily amenable to human or computational data mining techniques.

Drug repositioning is predominantly dependent on two principles: i) the "promiscuous" nature of the drug and ii) targets relevant to a specific disease or pathway may also be critical for other diseases or pathways [3,4]. The latter may be represented as a shared gene or feature (biological process, pathway, or phenotype) between a disease-disease, drug-drug, or a disease-drug. Based on this principle, some computational approaches (see recent review [5]) have been developed and applied to identify drug repositioning candidates ranging from mapping gene expression profiles with drug response profiles [6-12], to side-effect based similarities [13-15].

An increasing number of network-based methods built on "guilt by association" principle have also been used to identify drug repositioning candidates. For instance, Chiang and Butte computed disease-disease similarity network to identify drug repositioning candidates [16], while some other approaches used either drug-drug similarities [13,17] or both disease-disease and drug-drug similarities [18-20]. However, most of these approaches were either drug-centric or disease-centric and not "indications-centric". In other words, few studies have used a direct disease-drug-centric approach. While there have been studies using heterogeneous networks [17,21-24] for drug repositioning, to the best of our knowledge there have been no previous reports that (a) undertook a direct analysis of heterogeneous disease-drug network and (b) used network clustering-based approaches on heterogeneous networks to identify drug repositioning candidates.

In the current study, we built a gene and feature-based (shared biological processes, pathways, phenotype) disease and drug heterogeneous network and applied network clustering to identify drug repositioning candidates. We used two state-of-art network clustering approaches [25,26] to identify the modules of diseases-drugs. We validated the robustness of our methodology by removing ten percent of the edges and calculating the recovery rate of our predictions. Finally, we performed a literature and clinical trials data search to check for potential overlap of our discovered novel indications.

## Methods

### Disease-gene and drug-gene associations

Known disease-gene and drug-target associations were downloaded from KEGG Medicus (Feb, 2013), [27]. There were a total of 1301 diseases and 3613 drugs with at least one known gene association along with 1976 known indications (representing 364 diseases and 1066 drugs). To augment the drug targets, we also used drug-target data from DrugBank [28] using KeggDrug-DrugBank mappings (see Additional file 1 for a complete list of disease-genes and drug-targets).

### Generation of disease-disease, drug-drug, and disease-drug pairs based on shared genes or features

The nodes in our network are diseases and drugs while the edges represent either a shared gene or a shared feature (enriched biological process, pathway or phenotype). We first built a gene-based network where two nodes (disease or drug) are connected if they share a gene. We used *Jaccard *coefficient (see below) to measure the similarity between two nodes.

$$J\left(node1,node2\right)=\frac{\left|Genes_{node1}\cap Genes_{node2}\right|}{\left|Genes_{node1}\cup Genes_{node2}\right|}$$

**Figure 1:**
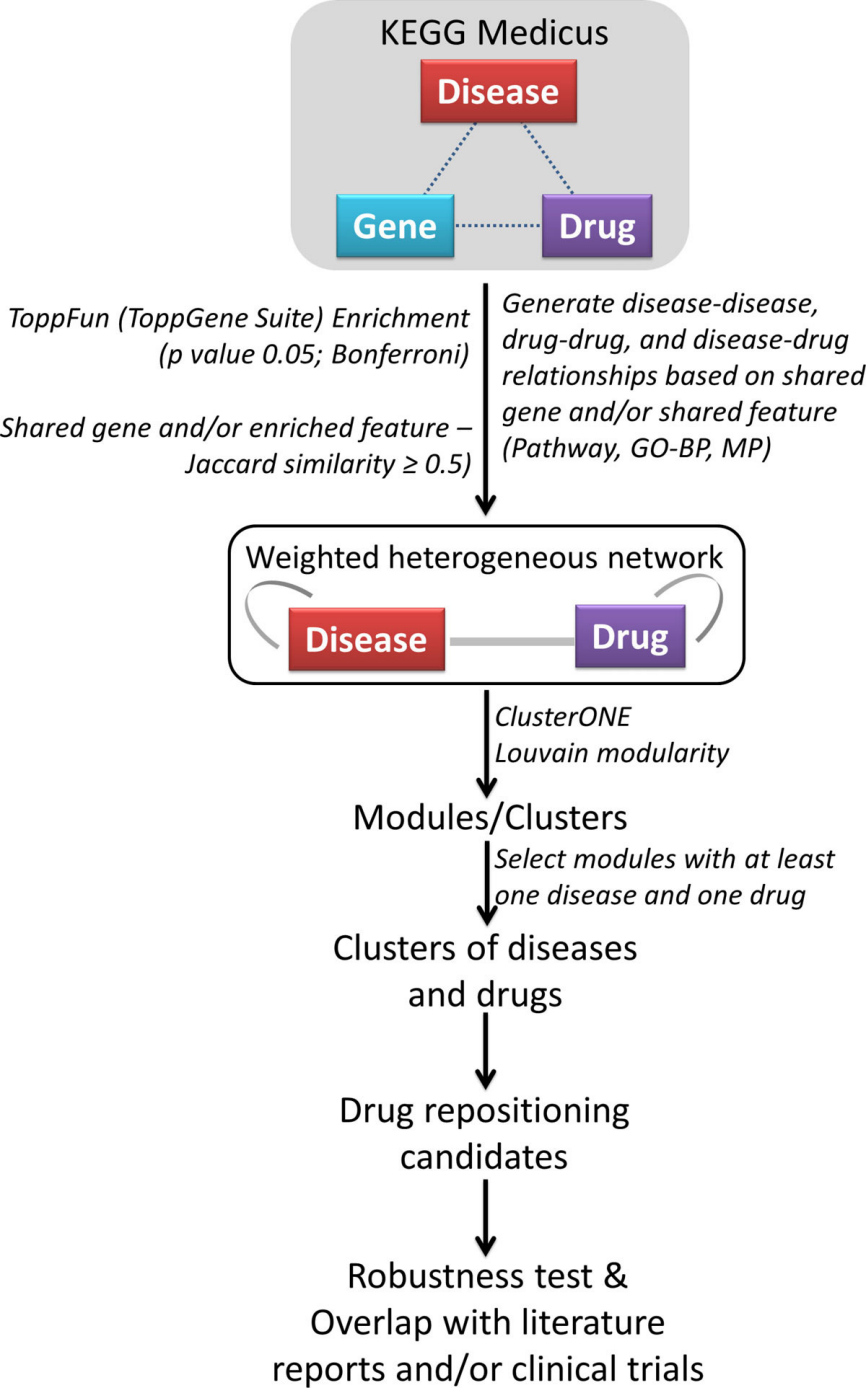
**Workflow for identifying drug repositioning candidates using gene- and feature-based disease and drug heterogeneous network**.

Because a disease or drug can be related to other disease or drug even if they do not share a gene, we further enhanced our network by adding edges that represent shared features (biological processes, pathways, and mouse phenotype). To do this, we first performed an enrichment analyses of each of the disease and drug using ToppFun application of the ToppGene Suite [29]. For each of disease and drug, we first computed the enriched biological processes, pathways, and mouse phenotype. We then built a feature-based network where nodes represent disease or drug while the edges represent shared enriched features (biological process, mouse phenotype and pathways; p-value ≤0.05 *Bonferroni *correction). We used *Jaccard *score to measure the feature similarity between each pair of the nodes. We thereby generated a list of disease-disease, drug-drug, and disease-drug pairs based on shared genes and/or enriched features (Figure 1).

### Graph clustering of weighted drug-disease heterogeneous network

We applied graph clustering to the weighted drug-disease heterogeneous network to extract densely connected clusters of diseases and drugs and mined them to extract potential candidates for drug repositioning. We used two state-of-art graph clustering algorithms, namely ClusterONE [26] and Louvain's modularity [25] for the module detection.

The Louvain method, in the first step, looks for "small" communities by optimizing modularity in a local way. In the second stage, it aggregates nodes of the same community and builds a new network whose nodes are the communities. These steps are repeated iteratively until a maximum of modularity is reached. This process naturally leads to hierarchical decomposition of the network and results in several partitions [25]. It measures the density of edges inside the community as compared to edges of inter-communities and is defined as:

$$Q=\frac{1}{2m}\underset{i,j}{\sum }\left[A_{i,j}-\frac{k_{i}k_{j}}{2m}\right]\delta \left(c_{i},c_{j}\right)$$

where $A_{i,j}$ represents the edge between node #160;i and  j, $k_{i}=\underset{j}{\sum }A_{i,j}$ is the sum of the weights of edges associated with node #160;i, $c_{i}$ is the community that node #160;i is assigned to, $\delta \left(u,v\right)$was 1 if u=v and 0 if otherwise and $m=\frac{1}{2}\underset{i\;j}{\sum }A_{ij}$. Although the partitioning seems like an approximate method and nothing ensures that the global maximum of modularity is attained, several tests have shown that it provides a decomposition in communities with modularity that is close to optimality [25]. The implementation is available as a plug-in in Gephi [30].

We also used another graph clustering approach, ClusterONE (Clustering with Overlapping Neighborhood Expansion) [26], to find the disease-drug modules. The cohesiveness of a cluster in ClusterONE is defined as follows:

$$f\left(V\right)=\frac{W^{in}\left(V\right)}{W^{in}\left(V\right)+W^{bound}\left(V\right)+P\left|V\right|}$$

where, $W^{in}\left(V\right)$ denotes the total weight of edges within a group of vertices *V*, $W^{bound}\left(V\right)$ denotes the total weight of edges connecting this group to the rest of the graph while $P\left|V\right|$ is the penalty term. We used ClusterONE because of its ability to identify overlapping cohesive sub networks in weighted networks and was shown previously to detect meaningful local structures in various biological networks [31,32]. We used the ClusterONE plug-in available in Cytoscape [33] for implementation.

## Results

### Analyses of known indications in disease-drug network

Starting with 1976 known indications (disease-drug pairs) from Kegg Medicus, we first filtered out diseases and drugs that do not have a known gene association in the Kegg database of disease genes and drug targets. This resulted in 1041 known indications representing 203 diseases and 588 drugs (Additional File 2). Using this data, we found that of the 1041 known indications (disease-drug pairs) only 132 pairs share at least one common gene (i.e., a disease-associated gene is also a drug target). We then checked if any of the known indications share a pathway. To do this, we used the disease-pathway and drug-pathway annotations from Kegg Medicus. While this also revealed that only 116 disease-drug pairs share a common pathway, what was surprising was that only 36 disease-drug pairs share both a pathway and a gene. This demonstrates that disease-drug relationships cannot be captured just through gene-centric approaches.

To analyze the characteristics of known indications further, we computed a distance measure between each of the known indication pairs in the human protein interactome (downloaded from NCBI's Entrez Gene [34]). We calculated the shortest path for all known indications (i.e., shortest path between a known disease and drug pair) in the protein interactions network using JUNG [35]. Of the 1041 known indications, we were able to compute the shortest paths for 1008 disease-drug pairs. For the remaining pairs, we were unable to compute the shortest paths because their encoded proteins were either absent in the interactome or were not reachable (e.g., a disease protein and drug target present in two different connected components of the protein interactome). The average distance between a disease-drug of known indications is 3.75 (median distance of 4), a finding concurred by previous reports [36]. These preliminary analyses, and our previous studies [37] with rare disease networks where we noted that the relationship between diseases cannot be fully captured by the genes network alone, motivated us to build a feature-based functional connectivity map between diseases and drugs.

### Disease-disease, drug-drug, and disease-drug pairs - edge pruning and weighted heterogeneous network generation

Using the disease-gene, drug-target, and the enriched features of diseases and drugs (based on functional enrichment analyses of diseases and drugs), we built a gene and feature-based network where nodes represent disease or drug while the edges represent shared gene and/or enriched features (biological process, mouse phenotype and pathways; p-value ≤0.05 *Bonferroni *correction). We used *Jaccard *score to measure the feature similarity between each pair of the nodes. In order to retain only edges that represent significant potentially significant relationships, we used a cutoff of 0.5 on *Jaccard *indexes across the four networks (gene-based and the 3 feature-based networks). Thus, the final network contained edges which were a union of pairs that passed the 0.5 *Jaccard *score threshold in each individual category.

Based on whether a pair of nodes (disease-disease, disease-drug, and drug-drug) shares genes or enriched features or both, we assigned weights to all the edges in the filtered pairs. For instance, a pair of nodes with a weighted edge of 1 indicates that they share either a gene or one of the three features whereas a weight of 4 indicates that the two nodes showed significant associations (sharing not only a gene but also the three features, namely, biological process, pathway, and phenotype). The resulting weighted heterogeneous network consisted of 657 disease nodes and 3489 drug nodes. The total number of edges in this network is 116493; 680 edges were between two diseases, 1626 were between a disease-drug and 114187 between two drugs (Additional File 3).

### Modularity analyses of the disease-drug network

We used two graph clustering algorithms to detect disease-drug modules in this weighted heterogeneous network of diseases and drugs. Using Louvain's method, we could identify 293 modules. Of these, 98 modules comprised nodes of both diseases and drugs. Using ClusterONE, we were able to partition the disease-drug heterogeneous network into 312 clusters (*p *value ≤ 0.05), of which, 110 clusters comprised both diseases and drugs (see Additional file 4 for a complete list of ClusterONE and Louvain method based modules) (Figure 1).

Using the ClusterONE and Louvain detected communities we generated all possible disease-drug combinations on a per cluster basis. We call these the "drug repositioning candidates". To test the robustness of these novel drug repositioning candidate pairs, we removed 10% of the edges at a time and calculated the recovery rate of our predictions in a repetitive manner. Briefly, in each run, we randomly removed 10% of edges from the heterogeneous weighted disease-drug network and performed graph clustering (both ClusterONE and Louvain methods) to detect the communities and extract drug repositioning candidate pairs. We repeated this for ten times and compared the drug repositioning candidates with those from the original network (before randomly removing the 10% edges). The average recovery rate in case of drug repositioning candidates generated by ClusterONE was ~95% while in case of Louvain clustering it was ~85%. This demonstrates that the drug repositioning candidates we have discovered are robust and that additional edge removal or addition will not affect the output significantly.

### Drug repositioning candidates and literature-based evaluation

**Figure 2:**
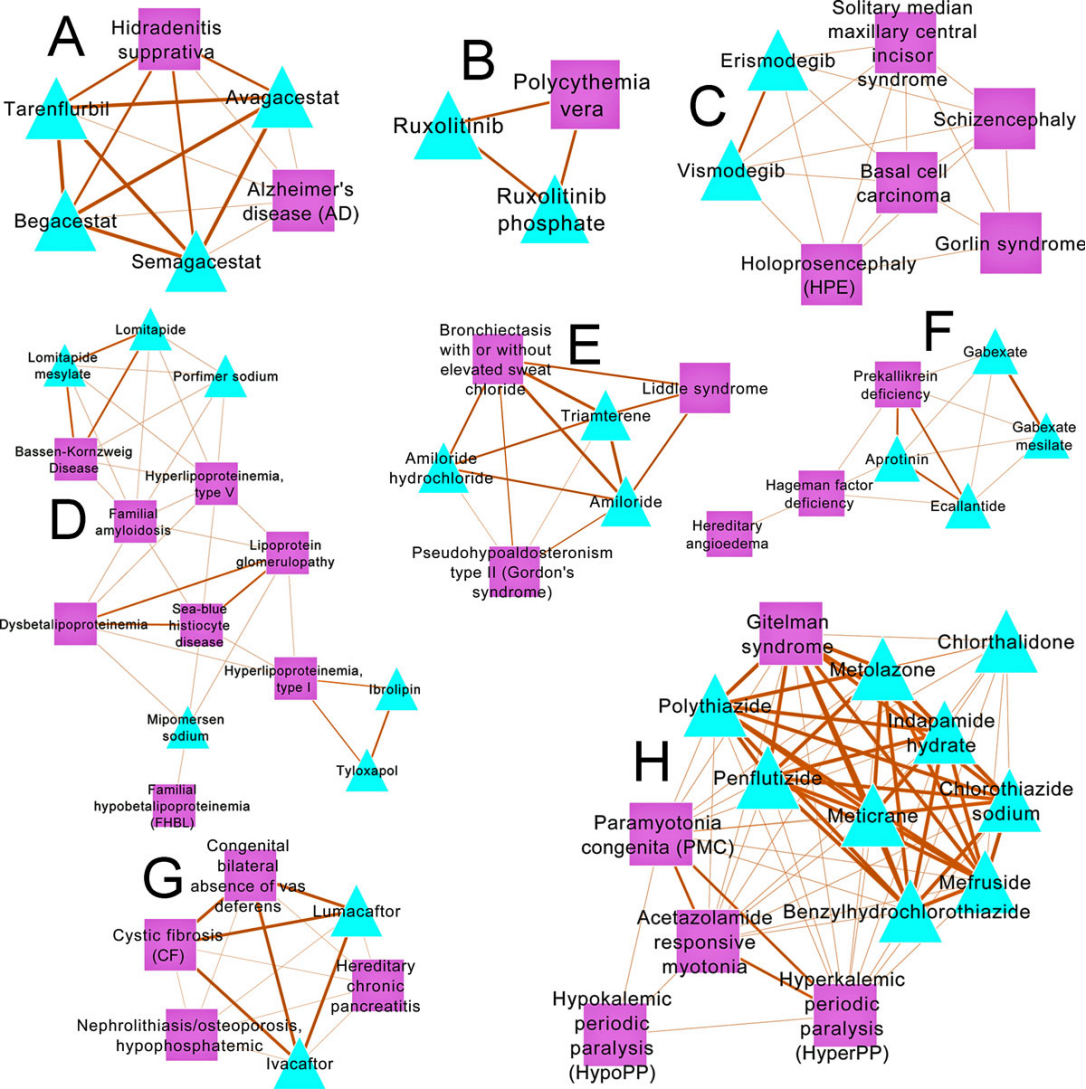
**Network of clusters harboring some of the drug repositioning candidates**. Square shaped nodes are diseases while the triangle nodes are drugs. The edge width is proportional to the number of genes/feature categories shared. Panels A though H represent modules of disease and drugs (based on ClusterONE or Louvain's modularity) which harbor some of the drug repositioning candidates that were backed by literature or clinical trials findings.

From the 98 clusters found by Louvain clustering, 11160 drug repositioning candidates (disease-drug pairs) were generated. In case of 110 ClusterONE-generated clusters, 2518 drug repositioning candidates were extracted. There were 2501 drug repositioning candidates (excluding 13 known indications) found by both of the clustering approaches (Additional file 5). We used these pairs to perform a literature-based and clinical trials search using CoPub [38] and a carefully designed PubMed search using NCBI's E-Utilities feature [39]. In the Figure 2 (panels A-H) we show the modules which contained drug repositioning pairs with literature evidence (see Table 1 for a list of drug repositioning candidate examples along that had either a literature-based and/or clinical trial-based evidence; See Additional File 6 for complete details including the PubMed IDs). In the following sections we discuss two case studies wherein our discovered drug repositioning candidates matched with those in clinical trials and literature.

**Table 1:** Examples of some of the drug repositioning candidates along with their count of PubMed references (see Additional file 6 for more details)

Disease ID	Disease Name	Drug ID	Drug Name	PubMed count
H00056	Alzheimer's disease	D09869	Avagacestat	5
H00056	Alzheimer's disease	D08869	Begacestat	5
H00056	Alzheimer's disease	D09377	Semagacestat	32
H00056	Alzheimer's disease	D09010	Tarenflurbil	21
H00079	Asthma	D09979	Tralokinumab	3
H00039	Basal cell carcinoma	D09992	Vismodegib	39
H00728	Brugada syndrome	D00303	Disopyramide	11
H00728	Brugada syndrome	D08215	Mexiletine	17
H00218	Cystic fibrosis	D09916	Ivacaftor	28
H00218	Cystic fibrosis	D10134	Lumacaftor	1
H00937	Familial male precocious puberty	D06247	Triptorelin	2
H00895	Gorlin syndrome	D09992	Vismodegib	5
H00242	Liddle syndrome	D07447	Amiloride	28
H00242	Liddle syndrome	D00386	Triamterene	4
H00012	Polycythemia vera	D09959	Ruxolitinib	19

### Vismodegib and Gorlin syndrome

**Figure 3:**
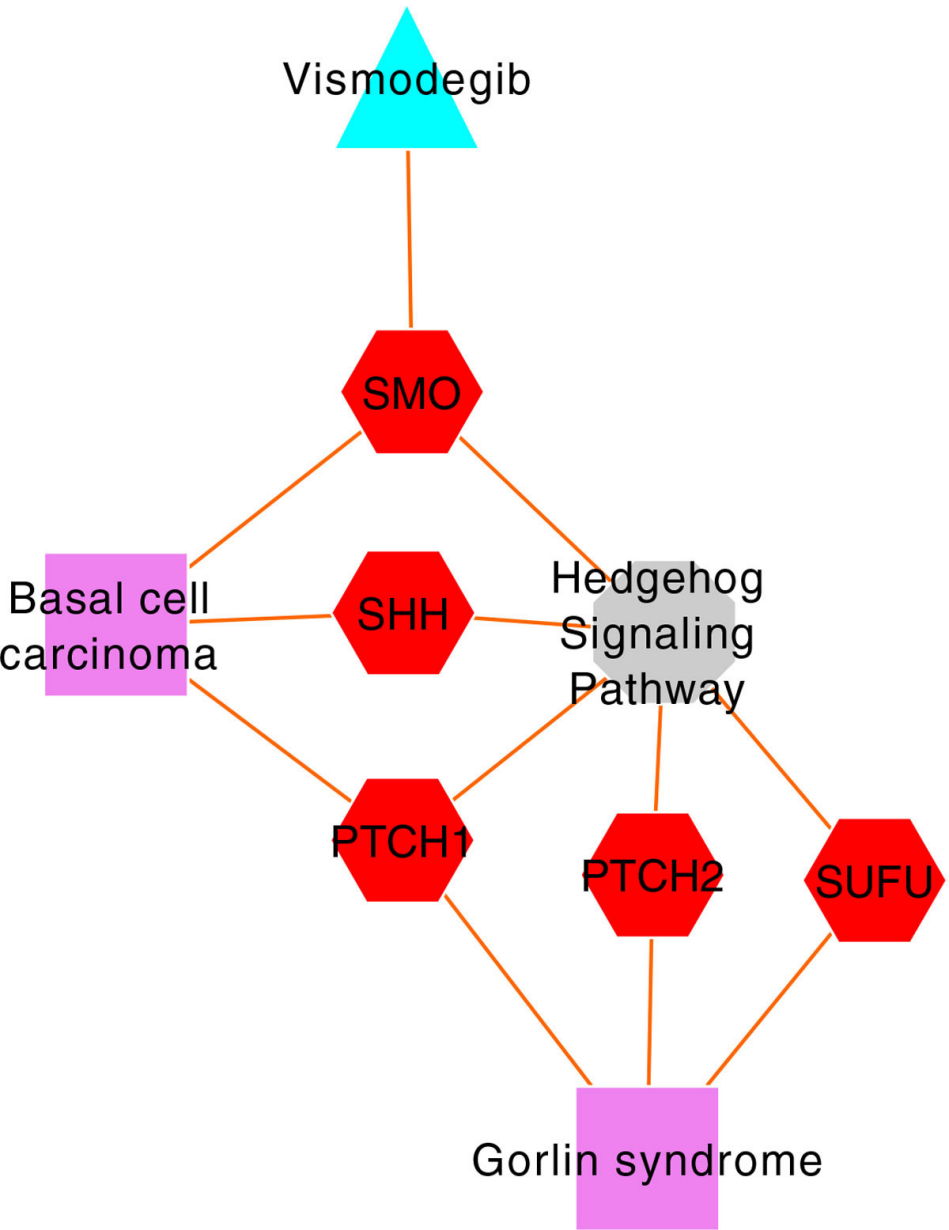
**Gene- and pathway-based connectivity map of vismodegib and Gorlin syndrome**. Drugs and diseases are represented as triangles and rectangles respectively. Genes are represented as hexagons while octagons represent pathways. Although vismodegib and Gorlin syndrome do not share a common gene, the hedgehog signaling pathway connects the drug and the disease.

Two of the drug repositioning candidates in our results that overlapped with the literature reports and clinical trials were derived from a cluster with drugs vismodegib and erismodegib and diseases basal cell carcinoma (BCC) and Gorlin syndrome. Interestingly, vismodegib, an oral inhibitor of the hedgehog pathway, is the first drug approved by the US Food and Drug Administration (FDA) for the treatment of locally advanced and metastatic BCC [40,41]. Additionally, another study reported the efficacy of vismodegib on patients with Gorlin syndrome (basal cell nevus syndrome), a rare autosomal dominant disorder in which those with the disease are prone to developing multiple BCCs at an early age [42] (clinical trial NCT00957229). In our analyses, vismodegib and Gorlin syndrome do not share a common gene but are still clustered together because of the pathway-based connectivity (hedgehog signaling pathway) (Figure 3). This demonstrates the utility of our approach in using feature-based heterogeneous networks to identify drug repositioning candidates.

### γ-secretase inhibitors, NSAID, Alzheimer's and Hidradenitis suppurativa

**Figure 4:**
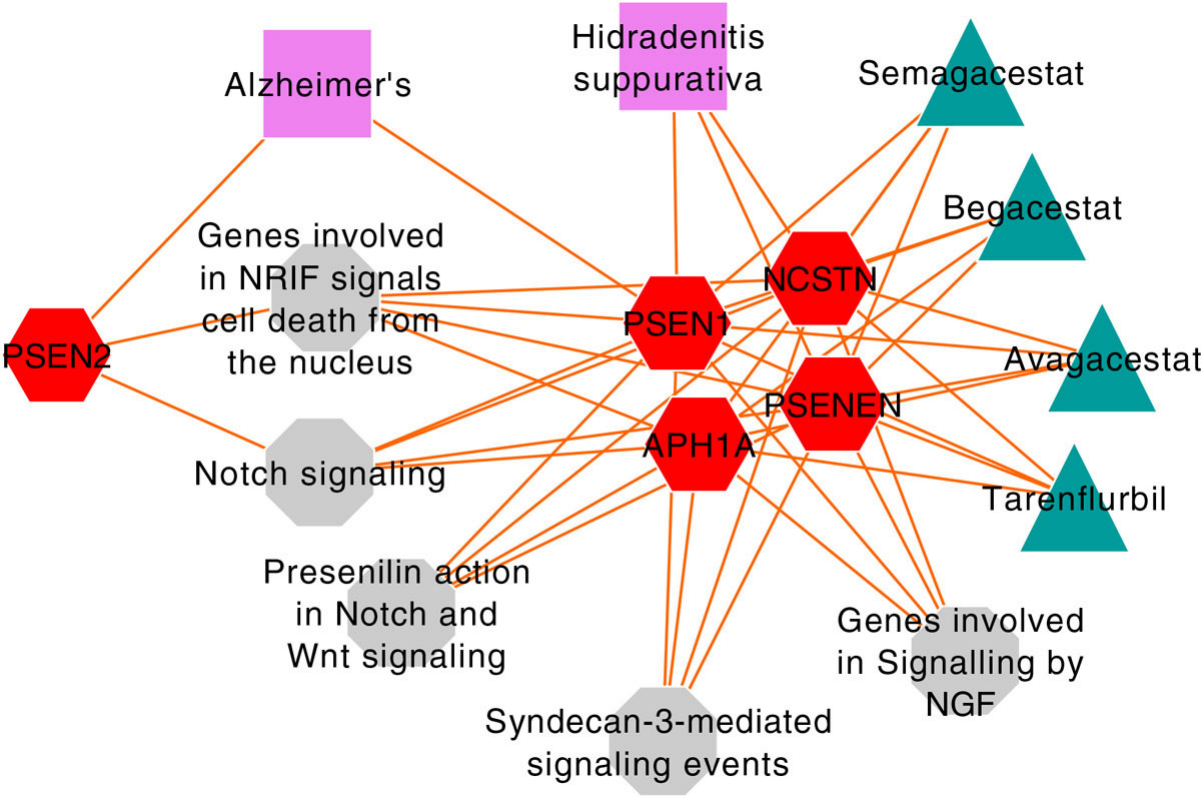
**Gene- and pathway-based connectivity map of Alzheimer's disease, γ-secretase inhibitors (semagacestat, begacestat, avagacestat), tarenflurbil, and hidradenitis suppurativa**. Triangular nodes represent drugs, rectangles represent diseases, hexagons represent genes and octagons represent pathways.

Another interesting set of examples in our study were related to Alzheimer's disease (AD) and γ-secretase inhibitors (avagacestat, semagacestat and begacestat) and NSAID (tarenflurbil or R-flurbiprofen) which have been shown as potent reducers of levels of β-amyloid (Aβ) [43-45]. In our study, AD and hidradenitis suppurativa (acne inversa) were clustered along with the γ-secretase inhibitors and tarenflurbil. Since several studies have implicated β-amyloid (Aβ) peptides in the etiology of Alzheimer's disease (AD) [46-48] and because Aβ is produced by the proteolytic cleavage of the amyloid precursor protein by β- and γ-secretase, γ-secretase inhibition is thought to have a therapeutic benefit for AD. However, all these drugs failed in phase III trials because they either worsened cognition and/or increased the risk of skin cancer. Although it is not known whether the adverse effects of γ-secretase inhibitors include hidradenitis suppurativa, our results show the clustering of γ-secretase inhibitors along with hidradenitis suppurativa. Interestingly, previous studies have shown that reduced γ-secretase and notch1 activity in mice cause a high frequency of skin cancer [49] and that hidradenitis suppurativa can be an allelic disorder of early-onset familial AD [50]. Indeed, the feature-based map of AD, hidradenitis suppurativa, γ-secretase inhibitors and tarenflurbil converge on the notch signaling pathway (Figure 4).

While the overlap of our discovered drug repositioning candidates with those under clinical trials (and literature evidences) demonstrates the utility of our approach, it also shows the limitations of computational approaches. In other words, while the computational approaches can provide potential candidates for drug repositioning, it may not be easy to foresee their failure in clinical trials. Nevertheless, the feature details (e.g., shared pathways, biological processes, phenotypes) our approach provides for the disease and candidate drug connectivity may not only help in understanding the molecular basis of side-effects but also make more informed decisions.

## Conclusions

Our approach to predict novel indications by representing disease-drug combinations as combinations of their molecular and mechanistic features, including biological processes, pathways, and phenotypes, not only led to the proposal of drug repositioning candidates but also allowed mechanistic insights into them. The robustness of our predictions and their overlap with those reported in the literature and clinical trials demonstrate that this approach can effectively identify new indications with the enriched feature patterns as an indicator for the mode of action. Although we have looked beyond the gene-based relationships, a limitation of this method is that it relies on the feature patterns enriched in diseases and drugs which themselves are generated using the genes associated with diseases or drugs. Thus, diseases and drugs that currently lack gene annotations are left out. Nevertheless, some of the discovered novel indications are far from being obvious and may also help in understanding the molecular basis of side effects. As Novac points out in a recent review [51], while it is too early to evaluate the success of repositioning efforts, the obvious candidates for repositioning may have already been exhausted. Thus, a much more thorough analysis and investment has to be done to reposition the rest of the candidates [51].

## Authors' contributions

CW and AJ conceived the study design which was coordinated by AJ. CW, RG, and AJ analyzed the data. BA participated in the interpretation and discussion of results. CW and AJ drafted the manuscript. All the authors have read and approved the final manuscript

## Supplementary Material

Additional file 1 **Disease-gene and drug-target data used in the study**. Click here for file

Additional file 2 **List of known indications (disease-drug pairs) used to analyze the distance metric in the protein interactome**. Click here for file

Additional file 3 **Details of heterogeneous network (disease-drug pairs) along with the edge details**. Click here for file

Additional file 4 **Details of clusters (ClusterONE and Louvain modularity)**. Click here for file

Additional file 5 **Complete list of drug repositioning candidates (from ClusterONE modules, Louvain modules, and those occurring in both)**. Click here for file

Additional file 6 **Examples of some of the drug repositioning candidates along with their PubMed references**. Click here for file

## Declarations

Funding for the publication fee and open access charge is from Division of Biomedical Informatics, Cincinnati Children's Hospital Medical Center, Cincinnati, OH, USA.

This article has been published as part of *BMC Systems Biology *Volume 7 Supplement 5, 2013: Selected articles from the International Conference on Intelligent Biology and Medicine (ICIBM 2013): Systems Biology. The full contents of the supplement are available online at http://www.biomedcentral.com/bmcsystbiol/supplements/7/S5.
